# Mangiferin Mitigates Gastric Ulcer in Ischemia/ Reperfused Rats: Involvement of PPAR-γ, NF-κB and Nrf2/HO-1 Signaling Pathways

**DOI:** 10.1371/journal.pone.0132497

**Published:** 2015-07-21

**Authors:** Magdy Mahmoud-Awny, Ahmed S. Attia, Mohamed F. Abd-Ellah, Hanan Salah El-Abhar

**Affiliations:** 1 Department of Pharmacology & Toxicology, October University, Cairo, Egypt; 2 Department of Microbiology & Immunologyology, Cairo University, Cairo, Egypt; 3 Department of Pharmacology & Toxicology, El-Azhar University, Cairo, Egypt; 4 Department of Pharmacology & Toxicology, Cairo University, Cairo, Egypt; Faculty of Pharmacy, Ain Shams University, EGYPT

## Abstract

Mangiferin (MF), a xanthonoid from Mangifera indica, has been proved to have antisecretory and antioxidant gastroprotective effects against different gastric ulcer models; however, its molecular mechanism has not been previously elucidated. Therefore, the aim of this study was to test its modulatory effect on several signaling pathways using the ischemia/reperfusion model for the first time. Animals were treated with MF, omeprazole (OMP), and the vehicle. The mechanistic studies revealed that MF mediated its gastroprotective effect partly via inducing the expression of Nrf2, HO-1 and PPAR-γ along with downregulating that of NF-κB. Surprisingly, the effect of MF, especially the high dose, exceeded that mediated by OMP except for Nrf2. The molecular results were reflected on the biomarkers measured, where the antioxidant effect of MF was manifested by increasing total antioxidant capacity and glutathione, besides normalizing malondialdehyde level. Additionally, MF decreased the I/R-induced nitric oxide elevation, an effect that was better than that of OMP. In the serum, MF, dose dependently, enhanced endothelial nitric oxide synthase, while reduced the inducible isoform. Regarding the anti-inflammatory effect of MF, it reduced serum level of IL-1β and sE-selectin, effects that were mirrored on the tissue level of myeloperoxidase, the neutrophil infiltration marker. In addition, MF possessed an antiapoptotic character evidenced by elevating Bcl-2 level and reducing that of caspase-3 in a dose related order. As a conclusion, the intimated gastroprotective mechanisms of MF are mediated, partially, by modulation of oxidative stress, inflammation and apoptosis possibly via the Nrf2/HO-1, PPAR-γ/NF-κB signaling pathways.

## Introduction

During gastric ulcer malady prosperity of upshots congregated to distress the balance between the destructive and the protective events. Oxidative stress is a key factor that engenders gastric ulcer and results in the overproduction of free radicals (FRs) [1]. The gastric ischemia/reperfusion (I/R) archetype mimics stress-induced gastric ulcer, in which case, elevated FRs, nitric oxide (NO), leucocyte adhesion molecule “E-selectin” [2], neutrophil infiltration [3], and redox imbalance [4] play a substantial role in its pathogenesis. Likewise, nuclear factor-kappa B (NF-κB), a redox-sensitive transcription factor, has a pivotal role in the I/R injury [5]. It regulates the expression of several genes associated with inflammation and cell injury, such as interleukin (IL) -1β, -6, cellular adhesion molecules and inducible nitric oxide synthase (iNOS). On the other hand, several factors mediate their gastroprotection against oxidative injury by alleviating the inflammatory response, these include peroxisome proliferator-activated receptor (PPAR)-γ [6, 7] and heamoxygenase-1 (HO-1) [8]. The latter is the inducible isoform of HO that responds to stresses, such as oxidative stress and it remains widely regarded as a protective mechanism against oxidative tissue injury [9].

One of the transcriptional regulators of HO-1 is the nuclear factor-E2-related factor-2 (Nrf2) [9], which is localized in the cytoplasm under normal conditions and interacts with Keap1 (the molecular sensor cysteine-rich protein Kelch-like ECH associating protein 1) to be rapidly degraded by the ubiquitin-proteasome pathway [10, 11]. Contrariwise, under oxidative stress conditions, Keap1 is oxidized and Nrf2 ubiquitination is lowered [11], liberating, thus, Nrf2 from Keap1. Nrf2 is then translocated into the nucleus to tether with the antioxidant response element of the target gene promoter [12, 13] causing immediate transactivation of the encoding genes. These genes include many enzymatic antioxidant and detoxifying genes, such as glutathione (GSH) peroxidase/reductase, HO-1, and GSH S-transferase [14].

Mangiferin (MF), a naturally occurring glucosylxanthone, possesses a gastroprotective effect *via* its antisecretory and antioxidant activities, as verified in different animal models of gastric ulceration [15], but not the I/R model, which is the goal of the current work. MF was reported to mediate its antioxidant activity at different levels of the oxidative sequence. It generates MF phenoxy radicals and binds to metal ions (Fe ^2+/3+^) in the form of a stable MF-iron complex that does not allow the generation of hydroxyl (OH˙) radicals and/or oxo-ferryl groups and scavenges lipid peroxy/alkoxy radicals, thereby, maintaining the cellular oxidation-antioxidant balance [16]. Despite the detailed cellular reactions described, however, the molecular signaling pathways has not been tackled before.

On the molecular level, the present aim was to assess the possible involvement of Nrf2/HO-1, PPARγ, and NF-κB signaling pathways in the gastroprotective effect of MF, besides other biomarkers to delineate the potential gastroprotective mechanisms using the hypoxia/ reoxygenation model.

## Materials and Method

### Animals

Male Wistar rats, weighing 180–220 g (Research Institute of Ophthalmology, Giza, Egypt) were kept on a 12h light/dark cycles, constant environmental conditions and were maintained on a proper diet chow and water *ad libitum*. Prior to experiment (24 hrs) all animals were kept individually in wide mesh bottom cages and fasted. Animals were handled according to the guidelines approved by the Animal Care and Use Committee of Faculty of Pharmacy, Cairo University, Cairo, Egypt (Permit Number: PT 575). All the surgical processes were performed under thiopental anesthesia, and all efforts were made to minimize suffering.

### Drugs

Mangiferin [MF] was purchased from Sigma-Aldrich Chemical Company (St Louis, MO, USA) and omeprazole [OMP] from Chemo S.A (Lugano, Switzerland). All other chemicals and reagents used were of analytical grade. MF and OMP were dissolved in saline to be injected intraperitoneally.

### Induction of gastric mucosal ischemia/ reperfusion (I/R) and treatments

The I/R model was performed according to the method previously described by Kotani et al. [17] with some modifications. Briefly, under thiopental anaesthesia (50 mg/kg, i.p), the rat coeliac artery was occluded for 30 min., after which reperfusion was allowed for 72 hrs by declamping the artery. Rats were allotted into 5 groups (n = 7–9); animals in the first group received saline and the celiac artery was handled without clamping to serve as the sham-operated control group. Rats in the following groups were subjected to I/R; the untreated (second) group, received saline and was denoted as the positive control (I/R), while the other 3 groups were treated, with MF (10 mg/ kg; MF_10_; third group), MF (20 mg/ kg; MF_20_; fourth group), and OMP (20 mg/ kg; OMP_20_; fifth group). All treatments were done 30 min. before operation and for 3 days after reperfusion.

### Histopathological examinations

Stomach, from three representative animals in each group, was immediately immersed in 10% formalin-saline, embedded in paraffin, and 5μm sections were prepared, stained with hematoxylin and eosin (H & E), and examined microscopically.

### Biochemical measurements

At the end of the reperfusion time and under deep ether anesthesia, blood was collected from the jugular vein to prepare sera, then animals were euthanized and the stomach was excised, opened along the greater curvature, and rinsed with an ice cold saline. The extent of gross mucosal damage (Ulcer Index) was assessed and expressed as the sum of ulcer lengths per stomach in mm [18]. Briefly, an illuminated magnifier (3x) was used to measure the length of the long lesions (mm) in the glandular part of the stomach, while the petechea lesions were counted and each five lesions were represented as 1 mm of ulcer.

About 50 mg of gastric mucosa was submerged overnight in RNA later solution, stored at -80°C till quantitative real-time PCR (qRT-PCR) quantification. The remaining mucosa was scrapped off and homogenized in ice-cold saline (MPW-120, Poland) using the maximum speed for 1 min. The homogenate was centrifuged at 5000 rpm for 5 min at 4°C and the resulting supernatants were kept in aliquotes and stored at -80°C till the determination of gastric parameters.

#### Estimation of homogenate/serum parameters using ELISA technique

The homogenate/serum levels of the following parameters were evaluated using the corresponding ELISA kits as shown in parentheses: IL-1β, sE-selectin (Abcam Inc., Cambridge, UK, Cat. No. ab100768 and ab171334, respectively), iNOS, eNOS (EIAab, Wuhan, China, Cat. No. E0837r and E0868r, respectively), B cell leukemia/lymphoma-2 (Bcl-2; USCN Life Science Inc., Wuhan, China, Cat. No. E90778Bo) and caspase-3 (Cusabio Biotech Co., Wuhan, China, Cat. No. CSB-E08857r). Each biomarker was processed according to the manufacturers' procedures provided. Serum total antioxidant capacity (TAC) was measured using the method of Benzie and Starin [19]. Briefly, ferric tripyridyltriazine (Fe^3+^-TPTZ) complex was reduced to the ferrous form by the antioxidants present in the sample at an acidic pH, to yield an intense blue color that can be monitored by measuring the change in absorbance at 593 nm. The change is directly related to the combined or total reducing power of electron donating non-enzymatic antioxidants present in the reaction mixture. The TAC level was expressed as μM/L.

#### Estimation of gastric activity/content of myeloperoxidase (MPO), malondialdehyde (MDA), reduced glutathione (GSH) and total nitric oxide (NOx)

The activity of MPO (U/gm), a marker of tissue neutrophil infitration, was assessed according to Bradley et al. [20]. The method is based upon measuring the hydrogen peroxide-dependent oxidation of o-dianisidine, catalyzed by MPO, which results in the formation of a compound exhibiting an increased absorbance at 460 nm. As an index of lipid peroxidation thiobarbituric acid reactive substances (TBARS), represented by MDA, was used to determine the oxidative damage, following the method of Mihara and Uchiyama [21]. The MDA-TBA adduct develops pink color, which was extracted by n-butanol and measured at two wave lengths, *viz*., 520 and 535nm. The method described by Ahmed et al. [22] was adopted to assess the non-protein sulfhydryl groups (mainly GSH) by reacting with Ellman’s reagent after precipitating the protein SH–groups. The reaction with Ellman’s reagent formed a stable yellow color of 5 mercapto-2-nitrobenzoic acid, which was measured colorimetrically at 412 nm (mg/gm).

Gastric mucosal NO production was estimated indirectly as nitrite/nitrate concentration according to the method of Miranda et al. [23], where vanadium trichloride was used to reduce nitrate into nitrite. The pink azo-dye produced by the reaction of nitrite with sulfanilic acid followed by the subsequent coupling with N-(1-naphthyl)-ethylenediamine was measured colorimetrically at 540 nm and NOx was expressed as μM/gm.

### Quantitative real-time (RT)-PCR

Total RNA was extracted from the gastric mucosa using Simply P Total RNA Extraction kit (BioFlux, Hangzhou, China). The purity of the obtained RNA was verified spectrophoto-metrically at 260/280 nm. Equal amounts of RNA (0.368 μg) were retrotranscribed into first-strand complementary DNA (cDNA) at 37°C for 50 min. using 200 U/μl M-MuLV reverse-transcriptase (SibEnzyme, Novosibirsk, Russia), 1 μl random hexamer (Qiagen, LRS Laboratories, Inc. Korea), and 0.1 M DTT in a 50 μL reaction mixture. To assess the expression of antioxidant and inflammation-associated target genes, RT-PCR was performed using SYBR green PCR Master Mix (Qiagen) as described by the manufacturer. Briefly, in a 25 μl reaction volume, 5 μl of cDNA was added to 12.5 μl of 2x SYBR green master mix and 2.5 μl (2.5 μM) of each primer. The sequences of primers were: PPAR-γ sense primer 5'-GCGGAGATCTCCAGTGATATC-3'; antisense primer 5'-TCA GCGACTGGGACTTTTCT-3'; NF-κB p65 sense primer 5'-TGCAGAAAGAAGACATTGAGGTG-3'; antisense primer 5'- AGGCTAGGGTCAGCGTATGG-3'; Nrf2 sense primer 5'-ATGGCC ACACTTTTCTGGAC-3'; antisense primer 5'-AGATGTCAAGCGGGTCACTT-3'; HO-1 sense primer 5'- CGTGCAGAGAATTCTGAGTTC-3'; antisense primer 5'- AGACG CTTTACGTAGTGCTG-3'; and glyceraldehyde-3 phosphate dehydrogenase (GAPDH) sense primer 5'-GGGCAGCCCAGAACATCA-3'; antisense primer 5'-TGACCTTG CCCACAGCCT-3'. PCR reactions included 15 min at 95°C for activation of HotStarTaq DNA Polymerase, followed by 45 cycles at 94°C for 15 sec (denaturing), 55°C for 30 sec (annealing), and 72°C for 30 sec (extension). The relative expression was calculated from the 2^-∆∆CT^ formula [24].

### Statistical analysis

Data were expressed as mean ± S.E.M of 7–9 animals. Statistical comparisons between means were carried out using one-way analysis of variance (ANOVA), followed by Student-Newman-Keuls test. The statistical significance of difference was considered at *P* < 0.05.

## Results

### Effect of MF and OMP on I/R-induced ulcer index

MF, dose dependently, prohibited the I/R-induced gastric injury, as presented by values of ulcer index (Table 1) and the effect of MF_20_ was comparable to that offered by OMP_20_.

**Table 1:** Effect of mangiferin and omeprazole on ulcer index in ischemia/reperfused rats. Mangiferin (10 & 20 mg/kg; MF_10_ & MF_20_) and omeprazole (20 mg/kg; OMP_20_) were administered intraperitoneally 30 min. before induction of ischemia/ reperfusion (I/R) and for 3 days after reperfusion. Values are means of 7–9 rats ± S.E.M; as compared with sham (*), I/R (^@^), MF_10_ (δ), and OMP (#) treated groups (one-way ANOVA followed by Student-Newman-Keuls multiple comparison tests), *P*< 0.05.

Groups	Ulcer index (mm)
**Sham**	0 ± 0
**I/R**	5 ± 0.4*
**I/R+MF_10_**	1.1± 0.08 ^@#^
**I/R+MF_20_**	0.3 ± 0.05 ^@δ^
**I/R+OMP_20_**	0.3±0.06 ^@^

### Effect of MF and OMP on the mRNA expression of antioxidant genes

**Fig 1:**
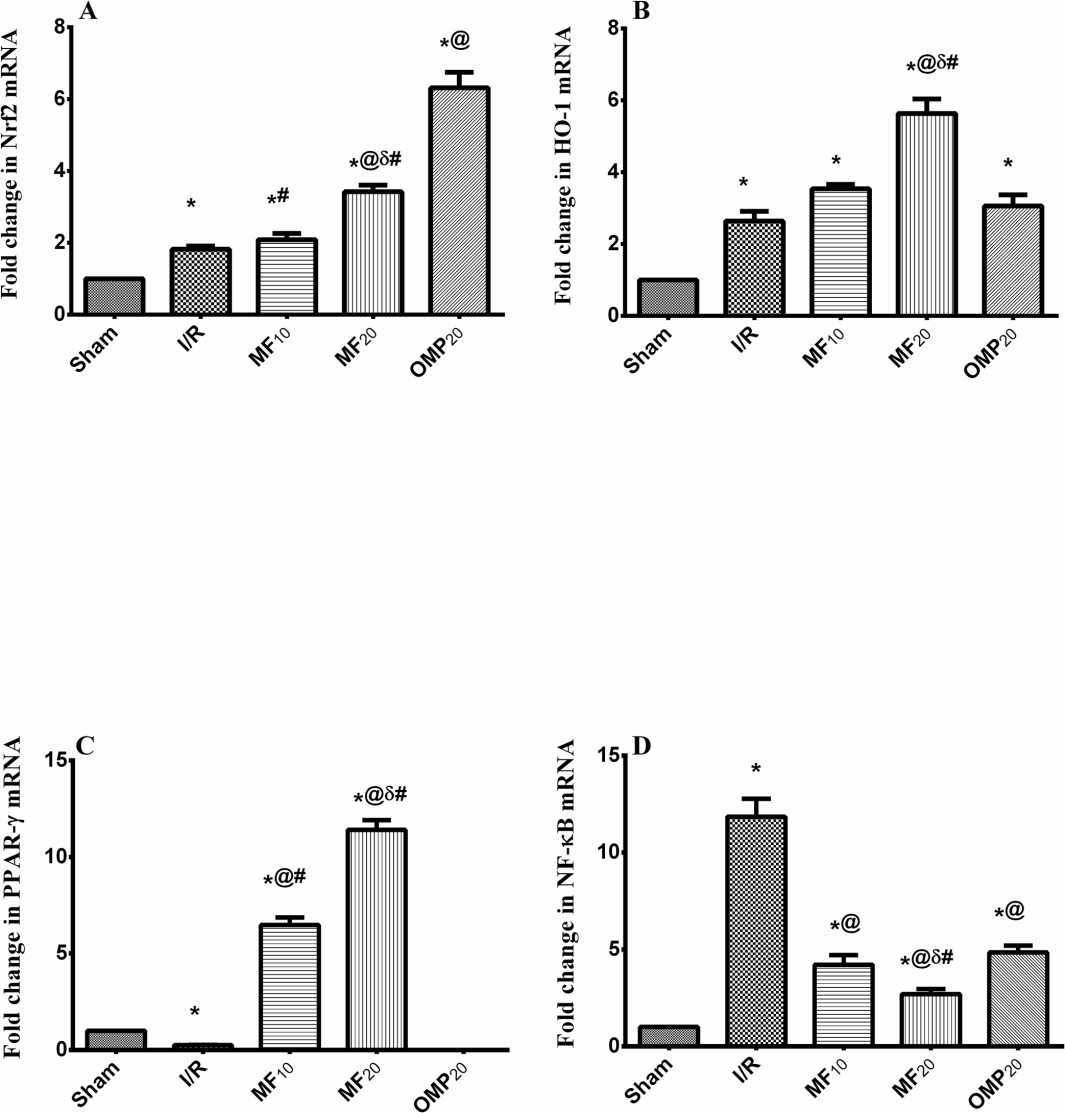
Effect of mangiferin and omeprazole on the mRNA expression of (A) Nrf2, (B) HO-1, (C) PPAR-γ, and (D) NF-κB genes in ischemia/reperfused rats. Mangiferin (10 & 20 mg/kg; MF_10_ & MF_20_) and omeprazole (20 mg/kg; OMP_20_) were administered intraperitoneally 30 min before induction of ischemia/reperfusion (I/R) and for 3 days after reperfusion. Values are means of 7–9 rats ± S.E.M; as compared with sham (*), I/R (^@^), MF_10_ (δ), and OMP (#) treated groups (one-way ANOVA followed by Student-Newman-Keuls multiple comparison tests) at *P*< 0.05.

As illustrated in Fig 1A and 1B, adaptive response against the I/R injury was evidenced in the upregulation of Nrf2 and HO-1 mRNA, which explains the reason behind the elevated GSH level in untreated I/R group. These mRNA upregulations were even more vivid in the treated groups, pointing, thus, to the antioxidant capacity of MF.

### Effect of MF and OMP on redox parameters

As presented in Tables 2 and 3 the I/R insult caused an imbalance in the oxidative/nitrosative status. A surge in MDA level (182%) and NOx production (187.6%), paralleled with 5.1 folds increase in iNOS, was perceived in the I/R untreated group. On the contrary, the I/R injury halved the beneficial eNOS and reduced the TAC (69.7%), compared to sham group. All these alterations were reverted by OMP, as well as MF, in a dose dependent manner. In almost all the parameters MF_20_ had the prominent effect. Regarding GSH, the I/R model, unexpectedly, elevated it by 32%, an effect that was further enhanced by the different drug regimens.

**Table 2:** Effect of mangiferin and omeprazole on redox parameters in ischemia/ reperfused rats. Mangiferin (10 & 20 mg/kg; MF_10_ & MF_20_) and omeprazole (20 mg/kg; OMP_20_) were administered intraperitoneally 30 min. before induction of ischemia/ reperfusion (I/R) and for 3 days after reperfusion. Values are means of 7–9 rats ± S.E.M; as compared with sham (*), I/R (^@^), MF_10_ (δ) and OMP (#) treated groups (one-way ANOVA followed by student-Newman-Keuls multiple comparison tests), *P*< 0.05. MDA (malondialdehyde), GSH (glutathione), and TAC (total antioxidant capacity).

Groups	MDA (nM/gm)	GSH (mg/gm)	TAC (μM/L)
**Sham**	35.2 ± 2.5	0.41 ± 0.03	153.3 ± 10.3
**I/R**	64 ± 1.8 *	0.54 ± 0.04 *	106.8 ± 6.4 *
**I/R + MF_10_**	45.2 ± 0.85*^@^	0.63 ± 0.05 *	192.7 ± 13.4*^@^
**I/R + MF_20_**	38.3 ± 3.3 ^@^	0.67±0.04*^@^	239.5±12.6*^@δ#^
**I/R + OMP_20_**	38.2 ± 3.1^@^	0.58 ± 0.02 *	193.7 ± 13 *^@^

**Table 3:** Effect of mangiferin and omeprazole on nitrosative stress parameters in ischemia/reperfused rats. Mangiferin (10 & 20 mg/kg; MF_10_ & MF_20_) and omeprazole (20 mg/kg; OMP_20_) were administered intraperitoneally 30 min before induction of ischemia/ reperfusion (I/R) and for 3 days after reperfusion. Values are means of 7–9 rats ± S.E.M, as compared with sham (*), I/R (^@^), MF_10_ (δ) and OMP (#) treated groups (one-way ANOVA followed by student-Newman-Keuls multiple comparison tests), *P*< 0.05. NOx (total nitric oxide), eNOS (endothelial nitric oxide synthase), and iNOS (inducible nitric oxide synthase)

Groups	NOx (μM/gm)	Enos (U/L)	iNOS (ng/mL)
**Sham**	142.5±11.6	25.6 ± 1.6	15.3 ±0.9
**I/R**	267.3±13.0*	14.1± 0.4 *	80.9±3.8*
**I/R + MF_10_**	177.8 ±9.1^@^	17.7±0.4*^@^	40.5±1.1*^@#^
**I/R + MF_20_**	192 ±10.2*^@^	23±0.4 *^@δ#^	28.1 ±0.8*^@δ^
**I/R + OMP_20_**	204.7±5.9 *^@^	18.2±0.5*^@^	29.1±0.7*^@^

### Effect of MF and OMP on the mRNA expression of PPAR-γ and NF-κB genes

As depicted in Fig 1C, animals with I/R injury revealed a significant decrease in the gastric expression of the anti-inflammatory PPAR-γ (25% of the sham level), with a marked increase in that of the proinflammatory factor NF-κB (11.9 fold) (Fig 1D). Interestingly, MF treated groups were able to counteract the I/R-related effects according to the dose level tested. OMP, on the other hand, showed a lesser effect than MF did on NF-κB, but did not alter the lessened PPAR-γ.

### Effect of MF and OMP on gastric inflammatory cytokines and neutrophils infiltration

Table 4 depicts the I/R-mediated gastric mischief, which aggravated the inflammatory biomarkers. It boosted the serum levels of IL-1β, sE-selectin, and gastric MPO activity by 5.2, 5.6, and 2.2 folds, respectively, as compared to the sham operated control group. All these alterations were significantly halted by the treatment with MF in a dose dependent manner. Nonetheless, the MF_20_ effects superseded that mediated by the reference standard OMP. These observations indicate that MF can modulate the inflammatory cytokines and neutrophils recruitment to mitigate I/R-induced injury.

**Table 4:** Effect of mangiferin and omeprazole on inflammatory cytokines, neutrophils infiltration and apoptotic biomarkers in ischemia/reperfused rats. Mangiferin (10 & 20 mg/kg; MF_10_ & MF_20_) and omeprazole (20 mg/kg; OMP_20_) were administered intraperitoneally 30 min before induction of ischemia/ reperfusion (I/R) and for 3 days after reperfusion. Values are means of 7–9 rats ± S.E.M, as compared with sham (*), I/R (^@^), MF_10_ (δ), and OMP (^#^) treated groups (one-way ANOVA followed by Student-Newman-Keuls multiple comparison tests) at *P*< 0.05. IL-1β (interleukin-1beta), MPO (myeloperoxidase), Bcl-2 (B cell leukemia/lymphoma2).

Groups	IL-1β (pg/mL)	sE-selectin (pg/mL)	MPO (U/gm)	Bcl-2 (ng/mL)	Caspase-3 (ng/gm)
**Sham**	11.4±0.5	1.9 ± 0.1	1.1± 0.1	61.3 ±1.2	6 ± 0.2
**I/R**	59.5±4.1 *	10.7 ± 0.3 *	2.4 ± 0.07 *	25.4 ±1.2*	41.1 ± 0.6 *
**I/R + MF_10_**	33 ±1.1 *^@^	8.8 ± 0.5 *^,@,#^	1.2± 0.07^@^	30.4 ±0.5*^@^	25 ±0.6 *^@^
**I/R + MF_20_**	15.5±0.4^@δ^	4.8 ± 0.3*^@δ#^	0.4±0.04*^@δ#^	45.8±1.3*^@ δ#^	14.5 ± 0.5 *^@ δ#^
**I/R + OMP_20_**	16.1± 0.4^@δ^	1.8 ± 0.03 ^@^	0.9± 0.01 ^@δ^	32.3 ±0.9*^@^	17.6 ±0.6 *^@ δ^

### Effect of MF and OMP on the apoptotic biomarkers

As presented in Table 4, the I/R insult triggered apoptosis of gastric mucosa as evidenced by the 6.85 folds increase of caspase-3 level. In the same context, I/R decreased the level of the anti-apoptotic marker Bcl-2 (59%). However, administration of MF, in a dose dependent manner, has halted these alterations significantly, effects that have surpassed those mediated by OMP.

### Effect of MF and OMP on gastric histopathological changes

**Fig 2:**
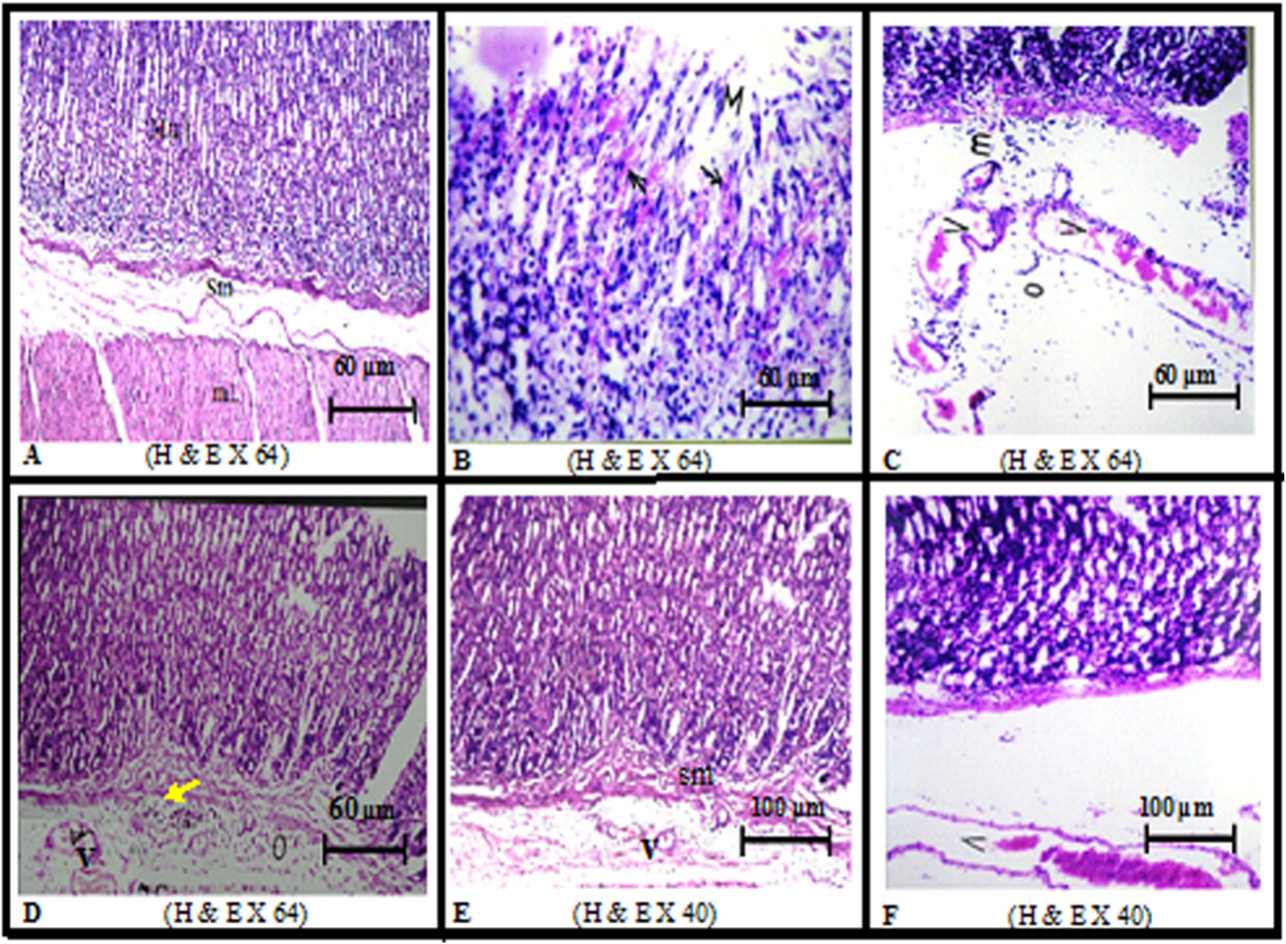
Representative photomicrographs of stomach mucosa. [A] Sham-operated rat section shows normal gastric mucosal (mu), submucosal (sm) and muscularis (mL) architecture. [B] Ischemia/reperfusion (I/R) sections show sloughed mucosa (m), juxtraposed with underlying haemorrhage (black arrow), [C] severe vascular congestion (V), focal inflammatory cells infiltration (m) and oedema (O) in submucosa. [D] MF_10_ section reveals only congestion in the submucosal blood vessels (V) with mild inflammatory cell infiltration (yellow arrow) and/or edema. [E] MF_20_ section, similar to sham-operated control, shows normal intact histological structure except for very mild vascular congestion (v) in the submucosa. [F] OMP_20_ section mimics that of MF_20_, but shows only congested blood vessels (V) in the submucosal layer.

The improving effects of the different drug regimens on the tested biomarkers were further confirmed by the histological findings presented in Fig 2. The figure illustrates the photomicrographs of stomach mucosa, which show [A] normal gastric mucosal (mu), submucosal (sm) and muscularis (mL) architecture in the sham-operated rat section. [B] I/R sections reveal sloughed mucosa (m), juxtraposed with underlying haemorrhage (black arrow), [C] severe vascular congestion (V), focal inflammatory cells infiltration (m), mainly as neutrophils, and oedema (O) in submucosa. [D] MF_10_ section divulges only congestion in the submucosal blood vessels (V) with mild inflammatory cell infiltration (yellow arrow) and/or edema. On the other hand, [E] MF_20_ section, similar to sham-operated control, shows normal intact histological structure, except for a very mild vascular congestion (v) in the submucosa; the MF_20_ effect superseded that of [F] OMP, where congested blood vessels (V) and oedema in the submucosal layer are still detected in the OMP sections.

## Discussion

Though previous studies have documented the antioxidant [15] and anti-inflammatory [25] capacities of MF, yet none has clarified the potential molecular pathways involved in mending the disturbed redox/inflammatory systems. Thus, in the present work we have verified the gastric mucosal expression of Nrf2/HO-1 to understand if this signaling pathway is involved in the MF antioxidant mechanism. Additionally, the study targeted the expression of NF-κB and PPAR-γ to explore also some of the possible MF anti-inflammatory molecular machinery.

Our signal transduction studies revealed that I/R has significantly upregulated the mRNA of Nrf2 and HO-1 pointing to a possible adaptive effect of the body against the I/R damaging insult; these findings mimic that of Pan and coworkers [26] in a retinal I/R model. Enhanced HO-1 protein expression, regulated by Nrf2 [27], can occur in response to oxidative stress [28] and inflammatory diseases and is widely accepted as a protective mechanism against oxidative tissue injury [9], facts that support our findings. MF, on the other hand, affirmed its antioxidant effect by elevating further the expression of Nrf2 and HO-1, results that were recorded previously in a hepatotoxic model [29]. To the best of our knowledge, this finding is the first direct evidence for a gastroprotective role of MF through the Nrf2/HO-1 antioxidant pathway in the I/R-induced ulcer model.

The antioxidant character of MF was further manifested herein by its ability to combat the I/R-induced elevation in lipid peroxides [30] and the decrease in TAC [31]. However, the effect of I/R and MF on GSH showed the same pattern observed in Nrf2 and HO-1 expression, where I/R caused a subtle, yet significant rise in GSH, an effect that was magnified further by MF [32]. This action, points to a possible compensatory response against I/R injury and links GSH with the Nrf2/HO-1 signaling pathway as supported hitherto by Das et al. [29]. The correlation between GSH and Nrf2 may be related to the molecular cross talk between the upregulated Nrf2 and the γ-glutamylcysteine ligase [10, 33], an enzyme that catalyzes the rate limiting step in GSH synthesis [34]. Additionally, HO-1 antioxidant role stems from its ability to catalyze the breakdown of the pro-oxidant heme into iron, biliverdin, and carbon monoxide [35]; biliverdin is then converted to bilirubin, which acts as an antioxidant against lipid peroxidation [36].

Besides mending the perturbed redox status, MF extended its effect to entail the nitrosative stress, as well. MF opposed the I/R effect on the levels of eNOS/iNOS, where it restored the activity of the constitutive enzyme eNOS [37] and decreased the noxious inducible isoform [38]. Therefore, it is expected that the elevated level of iNOS in the untreated group is responsible for the surplus level of NOx, which undermines gastric mucosal integrity *via* its interaction with superoxide anion and the formation of peroxynitrite [39]. The latter is a potent free radical that perturbs cell macromolecules through lipid peroxidation, direct mitochondrial impairment, inhibition of membrane Na^+^/K^+^-ATPase activity, and oxidative protein modification [40]. Contrariwise, the MF-mediated production of NOx can be driven from the restored eNOS, and not iNOS, supporting, thus, the antioxidant/free radical scavenging properties of MF [16]. The protective NO quenches free radicals with the consequent preservation of GSH in gastric mucosa; GSH acts both as a nucleophilic scavenger of superoxides and as a cofactor in the GSH peroxidase-mediated reduction of hydrogen peroxides [41].

The deleterious events that follow the I/R insult include increased release of proinflammatory mediators and recruitment of leukocytes, besides disturbing the oxidative/nitrosative machineries [42]. In the current study, MF validated its anti-inflammatory/ immune-modulatory effect, which was documented previously [25, 43], by inhibiting IL-1β [43], E-selectin [25] and neutrophil infiltration [44] in other different models. These effects can be linked partly to the MF-modulatory changes on the molecular level. MF induced the gene expression of PPAR-γ, along with the downregulation of the parent inflammatory transcription factor/mediator NF-κB, hindering, thus, the I/R effect. Earlier studies have reported that the I/R injurious effect is mediated partially *via* suppressing the PPAR-γ mRNA [7, 45], which is a transcription factor that acts as an influential pleiotropic regulator of anti-inflammation, antioxidant, and phagocyte-mediated cleanup processes. Apart from its direct genomic effect, PPARγ was found to interact negatively with other transcription factors as NF-κB, which underlies many aspects of the anti-inflammatory/immunomodulatory effect of PPARγ [25, 46], facts that tone with our findings. Furthermore, the enhanced expression of PPARγ was reported to inhibit the production of NOx/iNOS [47], offering, thus, an explanation for the MF-mediated inhibition in iNOS. PPARγ also impedes the macrophages-driven cytokines and leukocyte adhesion molecules, as seen herein, partly *via* suppressing the gene expression of NF-κB [25, 48]. Furthermore, MF was reported to reduce the adhesion of PMNs to the endothelium and guarded against their infiltration into gastric tissue [49], alongside the modulation of PPAR-γ and NF-κB expression. These findings support ours on E-selectin, iNOS, and MPO, as mentioned before. Additionally, the MF-induced HO-1 upregulation can rationalize the anti-inflammatory character of MF, *via* its byproduct carbon monoxide, which confers an anti-inflammatory property [50]. To that end, the experimental results of our study identify another gastroprotective mechanisms at both the molecular and cellular levels by which MF protected gastric mucosa against I/R-induced injury. These results corroborate with the findings of earlier studies in an experimental colitis model [51, 52], and pin down the anti-inflammatory action of MF.

Previously Wu et al. [53] reported that PPAR-γ overexpression also protects mitochondrial membrane potential and prevents apoptosis by upregulating the expression of the anti-apoptotic Bcl-2 family proteins. Similarly, carbon monoxide, the byproduct of HO-1 activity, confers an anti-apoptotic activity by the up-regulation of the anti-apoptotic molecule Bcl-2, and the down-regulation of the pro-apoptotic signal Bax [54]. Data of the current study demonstrated that I/R-induced gastric injury is associated also with apoptosis as evidenced by the marked reduction of Bcl-2 [55] and the elevation of caspase-3 [56], while MF reverted the I/R-induced apoptosis by a dose dependent elevation of Bcl-2 level, and reduction of caspase-3 level. The MF anti-apoptotic effect coincides with the results of Ghosh et al. [38] and may be attributed to the MF-mediated upregulation of PPAR-γ and/or HO-1 mRNA levels.

All these findings were further reflected on the histological changes; the I/R insult caused shedding of the epithelial protective layer and resulted in an underlying haemorrhage with a marked congestion of blood vessels along with inflammatory cells infiltration in the lamina propria, an effect which designates blood flow cessation that was accompanied by oedema. The congestion of blood vessels may result from the elevation of the vasoconstrictor endothelin-1 in gastric tissue [57], increased leukocyte infiltration [58], and/or decreased activity of eNOS as documented in the current study. Accordingly, I/R was not only accompanied by free radical formation, but also with a clear decrease in the levels of endogenous antioxidants and increased infiltration of inflammatory cells, alterations that were hindered by MF, especially at the high dose level, where normal histological structure was observed.

Regarding the reference drug OMP, which exerts its gastroprotective effect by its antioxidant, antiapoptotic [59], and anti-inflammatory [60] actions, beyond acid suppression [61], we delineated herein the molecular events, which contribute to these effects. In the present study, we documented that OMP antioxidant effects, evidenced by inhibiting lipid peroxidation and increasing the defense biomarkers, are mediated *via* the Nrf2/HO-1 signaling pathway. OMP also inhibited the proinflammatory cytokine IL-1β, as well as neutrophil adhesion and infiltration as represented by reducing sE-selectin level and MPO activity, respectively. Although OMP downregulated the expression of NF-κB, yet it could not combat the I/R effect on PPAR-γ, suggesting that the anti-inflammatory/ immune-modulatory effect of OMP stems from suppressing NF-κB gene expression, and possibly other mechanisms, but not that of PPAR-γ.

Although the effect of OMP on Nrf2 expression exceeded that of MF_20_, yet the opposite was obvious on HO-1 and GSH, a discrepancy that may be linked to their effects on NF-κB. The latter was proved to act as a negative regulator of the Nrf2 pathway by competing with it for binding to the transcriptional co-activator CREB-binding protein (CBP) and also to promote the binding of the co-repressor histone deacetylase 3 (HDAC3) to the antioxidant response element (ARE) [62]; these effects may retard the Nrf2 mediated transcription of its downstream targets.

Furthermore, the antiapoptotic property of OMP relied not only on its ability to increase the level of the antiapoptotic protein, Bcl-2 or to decrease the level of the apoptotic marker, caspase-3, as detected in the present study, but also on the reduction of oxidative DNA damage [59].

Finally, one can conclude that the gastroprotective effect of MF, especially at a dose of 20 mg/kg, may be ascribed to the activation of the Nrf2/HO-1 antioxidant pathway, the PPAR-γ anti-inflammatory pathway *via* downregulating NF-κB, and to increasing the antiapoptotic protein Bcl-2 and decreasing caspase-3. All these mechanisms finally maintain normal gastric mucosal barrier integrity.
